# Genetic Characteristics of Patients with Young-Onset Myelodysplastic Neoplasms

**DOI:** 10.3390/jcm12247651

**Published:** 2023-12-13

**Authors:** Hyun-Young Kim, Keon Hee Yoo, Chul Won Jung, Hee-Jin Kim, Sun-Hee Kim

**Affiliations:** 1 Department of Laboratory Medicine and Genetics, Samsung Medical Center, Sungkyunkwan University School of Medicine, Seoul 06351, Republic of Korea; hyuny.kim@samsung.com (H.-Y.K.); heejinkim@skku.edu (H.-J.K.); 2 Department of Pediatrics, Samsung Medical Center, Sungkyunkwan University School of Medicine, Seoul 06351, Republic of Korea; keonhee.yoo@samsung.com; 3 Division of Hematology-Oncology, Department of Medicine, Samsung Medical Center, Sungkyunkwan University School of Medicine, Seoul 06351, Republic of Korea; chulwon1.jung@samsung.com

**Keywords:** myelodysplastic neoplasm, young onset, germline predisposition, somatic mutation, U2AF1 mutation

## Abstract

Myelodysplastic neoplasm (MDS) is a heterogeneous group of myeloid neoplasms affected by germline and somatic genetic alterations. The incidence of MDS increases with age but rarely occurs at a young age. We investigated the germline and somatic genetic alterations of Korean patients with young-onset MDS (<40 years). Among the thirty-one patients, five (16.1%) had causative germline variants predisposing them to myeloid neoplasms (three with *GATA2* variants and one each with *PGM3* and *ETV* variants). We found that PGM3 deficiency, a subtype of severe immunodeficiency, predisposes patients to MDS. Somatic mutations were identified in 14 patients (45.2%), with lower rates in patients aged < 20 years (11.1%). Nine (29%) patients had *U2AF1* S34F/Y mutations, and patients with *U2AF1* mutations showed significantly worse progression-free survival (*p* < 0.001) and overall survival (*p* = 0.006) than those without *U2AF1* mutations. A *UBA1* M41T mutation that causes VEXAS syndrome was identified in a male patient. In conclusion, a germline predisposition to myeloid neoplasms occurred in ~16% of young-onset MDS patients and was largely associated with primary immunodeficiencies, including GATA2 deficiency. Furthermore, the high frequency of somatic *U2AF1* mutations in patients with young-onset MDS suggests the presence of a distinct MDS subtype.

## 1. Introduction

Myelodysplastic neoplasm (MDS) is a heterogeneous group of myeloid neoplasms characterized by ineffective hematopoiesis, dysplastic hematopoietic cells, peripheral blood cytopenia, and a risk of progression to acute myeloid leukemia (AML) [1]. MDS is characterized by distorted hematopoietic stem cell function, inflammatory and innate immune dysregulation, deregulated apoptosis, and multiple genomic events, which result in heterogeneous clinical symptoms and outcomes [2]. The incidence of MDS increases with age, starting to rise rapidly after the age of 50 years, and is most prevalent in those aged 70–80 years [3,4,5]. Conversely, MDS rarely occurs in individuals under the age of 40, with an incidence rate of 0.14 per 100,000 in the United States [3]. In Korea, the rates of 0.48 and 0.39 per 100,000 individuals have been reported for males and females under 35 years of age, respectively [5]. 

Both germline and somatic genetic alterations contribute to the development of MDS [6]. Furthermore, these genetic alterations in patients with MDS play a crucial role in the clinical phenotypes, prognosis, and responses to treatment, making them crucial for predicting the patient’s clinical course and optimizing treatment and management [7]. Somatic alterations are found in approximately 78–90% of patients with MDS and primarily occur in genes associated with DNA methylation (*TET2*, *DNMT3A*, *IDH1/IDH2*), chromatin modification (*ASXL1*, *EZH2*), RNA splicing (*SF3B1*, *SRSF2*, *U2AF1*), and DNA damage pathway (*TP53*) [2,7,8]. However, due to the low prevalence of MDS in young age groups, most of the patients included in studies on somatic mutations in MDS are older, and it is not well understood whether somatic mutations occurring in young patients differ from those occurring in older patients. 

Germline predisposition to myeloid neoplasms has been reported in relation to germline alterations in *CEBPA*, *DDX41*, *TP53*, *RUNX1*, *ANKRD26, ETV6*, *GATA2*, and *SAMD9/SAMD9L* as well as alterations in genes associated with bone marrow failure syndromes (Fanconi anemia, dyskeratosis congenita and related telomere biology disorders, Diamond–Blackfan anemia, and Shwachman–Diamond syndrome) [9,10,11,12]. The causative genes and their frequencies in these germline predispositions have been reported to vary across different studies, and additional causative genes are still being discovered [13,14,15]. In particular, bone marrow failure syndromes that overlap clinically with MDS share overlapping clinical features with primary immunodeficiencies, and a recent study reported that approximately 17% of patients with bone marrow failure have underlying primary immunodeficiencies [16]. Furthermore, germline predisposition genes exhibit age-related differences, with germline *DDX41* variants observed in approximately 2.4–3.8% of patients with myeloid neoplasms and are prevalent in older age [17,18,19]. In contrast, genes other than *DDX41* are known to cause myeloid neoplasms in younger age groups, and there may be racial differences (for instance, between European and East Asian) in the distribution and frequency of genes that cause germline predisposition. 

In this study, we aimed to investigate the germline and somatic genetic characteristics of young-onset Korean patients with MDS and to examine the clinical and prognostic features associated with genetic factors.

## 2. Materials and Methods

### 2.1. Patients

We retrospectively evaluated 31 patients with young-onset MDS for whom specimens for genetic testing were available, diagnosed between 2007 and 2020 at our institution; their characteristics are summarized in Table 1. Young-onset MDS was defined as an onset age of less than 40 years, and the median age of the patients was 24 years (range, 0–39 years). All patients were diagnosed or revised according to the 2016 and 2022 World Health Organization (WHO) classification of myeloid neoplasms [11,20]. Clinical and laboratory information of the patients, including complete blood cell count (CBC), bone marrow (BM) examination, and cytogenetic and molecular genetic studies, were obtained from electronic medical records. Cytogenetic risk stratification was based on the revised international prognostic scoring system (R-IPSS) [21]. As a control group for somatic mutations detected in patients with young-onset MDS, we used targeted NGS data from 50 patients aged 40 years or older who were diagnosed with MDS and underwent targeted NGS testing for 38 genes (Supplementary Table S1) between November 2018 and October 2021. This study was approved by the Institutional Review Board of the Samsung Medical Center, Seoul, Korea (SMC IRB No. 2021-12-150). 

### 2.2. Molecular Genetic Study

Genomic DNA was extracted from bone marrow aspirates at the time of diagnosis using the Wizard Genomic DNA Purification Kit (Promega, Madison, WI, USA) or the QIAamp DNA Blood Mini Kit (Qiagen, Venlo, The Netherlands), according to the manufacturer’s instructions. Library preparation was performed using a G-Mendeliom panel (Celemics, Seoul, Republic of Korea) containing 5857 disease-related genes, and sequencing was performed on the DNBSEQ-G400 platform (MGI Tech Co., Ltd., Shenzhen, China), with an average sequencing coverage of 160x. Reads were aligned using the BWA-MEM tool (version 0.7.17) to the human genomic reference sequence GRCh37/hg19, variant calling was performed using the GATK package (v4.1.8), and annotation was performed using VEP101 (VariantEffect Predictor) and dbNSFP v4.1. Each variant was annotated with a population database (Genome Aggregation Database (gnomAD), Exome Sequencing Project (ESP), Korean Reference Genome Database (KRGDB)) and disease databases (ClinVar, Human Gene Mutation Database (HGMD), Online Mendelian Inheritance in Man (OMIM), and Catalogue of Somatic Mutations in Cancer (COSMIC)). In silico analyses were performed using SIFT, PolyPhen-2, MutationTaster, and SpliceAI. To identify germline variants predisposing patients to myeloid neoplasms, we performed a variant analysis of 524 genes that were possibly associated with myeloid neoplasm predisposition (Supplementary Table S2). We considered variants with a variant allele frequency (VAF) of ≥40% as presumed germline variants in these genes, except recurrent variants, reported 10 times or more in COSMIC. We assessed their pathogenicity according to the American College of Medical Genetics and Genomics (ACMG) and Association for Molecular Pathology (AMP) guidelines for sequence variants [22], and referred to pathogenic and likely pathogenic variants as causative variants. Also, we assessed somatic mutations classified as clinically significant (Tier I/II), following the 2017 AMP, American Society of Clinical Oncology (ASCO), and College of American Pathology (CAP) somatic variant guideline [23], in 38 genes where somatic mutations commonly occur in myeloid neoplasms (Supplementary Table S3). All clinically relevant variants were visually inspected using Integrative Genomic Viewer [24].

### 2.3. Germline Testing

Germline testing was performed for presumed causative germline variants using bone marrow aspirates or peripheral blood in remission. Direct sequencing was performed using primers specific to the target variant on an ABI Prism 3130xl Genetic Analyzer using the BigDye Terminator Cycle Sequencing Ready Reaction Kit (Applied Biosystems, Foster City, CA, USA).

### 2.4. Statistical Analysis

Categorical variables were compared using the chi-square test or Fisher’s exact test, as appropriate, and continuous variables were compared using the Mann–Whitney U test. Progression-free survival (PFS) was determined from the time of initial diagnosis to disease progression or the last follow-up, and overall survival (OS) was determined from the time of initial diagnosis to death or the last follow-up. Survival analysis was performed using Kaplan–Meier plots, and differences in survival were compared using the log-rank test. All statistical analyses were performed using SPSS version 27 software (IBM Corp., Armonk, NY, USA). A *p*-value < 0.05 was considered statistically significant.

## 3. Results

### 3.1. Germline Variants Predisposing to Myeloid Neoplasms in Young-Onset MDS

Among the thirty-one patients, five (16.1%) had causative germline variants predisposing them to myeloid neoplasms in the *GATA2*, *PGM3*, and *ETV6* genes (Table 2), and all of these identified variants have been previously reported [25,26,27,28,29,30]. *GATA2* was the most common causative gene (9.7%), and three distinct *GATA2* heterozygous variants were identified in three male patients aged 22–28 years. Among them, one patient (R361H) had warts in the nostrils; another (K390del) had prolonged and severe infections, ultimately leading to uncontrolled pneumonia and death; and the third patient (Y141*) had previously been found to have a hypocellular marrow. Cytogenetic abnormalities, including trisomy 8 or 7q deletions, were found in two patients with *GATA2* variant. A homozygous splicing variant (c.871+5G>A) of *PGM3* was found in a 3-year-old girl. She had experienced recurrent pneumonia since early childhood. At the time of admission, she presented with anemia and severe neutropenia accompanied by eosinophilia, and BM examination revealed hypocellular marrow along with dysplasia in granulocytes and megakaryocytes. Given that the previous microarray analysis confirmed a heterozygous 6q14.1q14.3 microdeletion encompassing *PGM3* in this patient, we concluded that the patient carried compound heterozygous variants consisting of a heterozygous *PGM3* deletion and a heterozygous splicing variant. Finally, a heterozygous nonsense variant (R359*) of *ETV6* was identified in a 28-year-old male, which was clinically consistent with a previous history of thrombocytopenia. 

### 3.2. Somatic Mutations and Prognostic Significance of U2AF1 Mutation in Young-Onset MDS

Somatic mutations were identified in 14 patients (45.2%) (Table 3), and patients younger than 20 years had a lower somatic mutation rate than those older than 20 years (11.1% vs. 59.1%; *p* = 0.015). The presence of somatic mutations was not significantly different between patients with and without an underlying germline predisposition (60% vs. 42.3%, *p* = 0.467). Notably, nine (29%) patients had *U2AF1* mutations. All *U2AF1* mutations were missense mutations occurring in codon 34, with a median VAF of 42% (range, 37–52%). Specifically, S34F and S34Y were observed in five (16.1%) and four (12.9%) patients, respectively, and there was no difference in VAF among the mutations (42% vs. 43.2%; *p* = 0.806). In contrast, mutations in other genes, including *ASXL1*, *DNMT3A*, *SF3B1*, *STAG2*, and *TP53*, were observed at lower frequencies, ranging from once to twice. 

**Figure 1:**
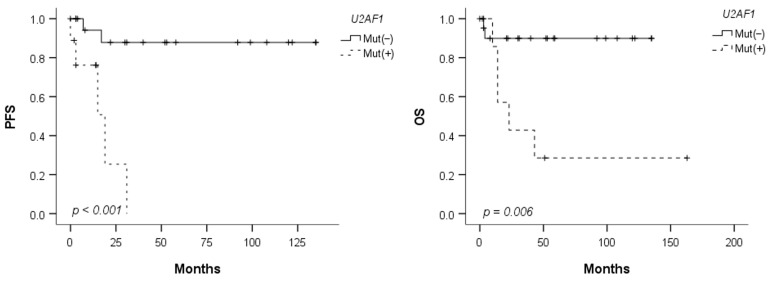
Progression-free survival (PFS) and overall survival (OS) according to the *U2AF1* mutation status.

When compared with a cohort of 50 patients with late-onset MDS (≥40 years), *U2AF1* mutations were significantly more frequent in patients with young-onset MDS (29% vs. 6%; *p* = 0.008), whereas other somatic mutations were observed at much lower frequencies (Table 4). In the survival analysis stratified by *U2AF1* mutation status, patients with *U2AF1* mutations showed inferior PFS and OS compared to those without *U2AF1* mutations (*p* < 0.001 and *p* = 0.006, respectively) (Figure 1). However, no significant differences were observed in the other clinical and laboratory findings based on the presence of *U2AF1* mutations (Supplementary Table S4).

### 3.3. Clinical Course of a Case with Somatic UBA1 Mutation

**Figure 2:**
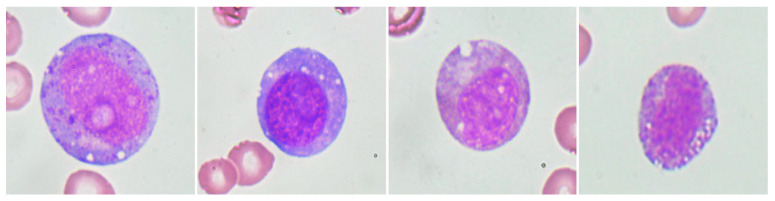
Cytoplasmic vacuolation of myeloid precursor cells observed in the bone marrow of a patient with somatic *UBA1* mutation.

Interestingly, the diagnostic marker for VEXAS syndrome, *UBA1* M41T mutation (VAF, 83.3%), was identified in a 38-year-old male patient, and we reviewed his clinical disease course. Three years prior to the diagnosis of MDS, he developed a progressive erythematous maculopapular rash accompanied by fever, myalgia, and tenderness, along with auricular chondritis and polyarthritis. At that time, the CBC showed Hb 10.6 g/dL, WBC 3.73 × 10^9^/L, and a platelet count of 167 × 10^9^/L. A BM examination performed due to a history of leukopenia showed normocellular marrow with no evidence of dysplasia but accompanied by granulocytic hyperplasia. Subsequently, the patient was diagnosed with relapsing polychondritis and treated with prednisolone and/or cyclosporine. During follow-up, as cytopenia worsened, the second BM examination was performed three years later. CBC showed Hb 9.5 g/dL, WBC 3.08 × 10^9^/L, and platelets 128 × 10^9^/L. BM examination revealed a hypercellular marrow with trilineage dysplasia, leading to the diagnosis of MDS with multilineage dysplasia according to the 2016 WHO classification. While receiving conservative treatment for MDS, the cytopenia deteriorated significantly. Six years after the diagnosis of MDS, the patient underwent allogeneic hematopoietic stem cell transplantation, and disease-related symptoms improved with complete engraftment. In the pre-transplant bone marrow examination, characteristic findings of VEXAS syndrome, specifically cytoplasmic vacuolation of myeloid precursor cells, were observed (Figure 2).

## 4. Discussion

This study identified diverse genetic characteristics in young-onset MDS patients from germline and somatic perspectives. The identification of germline variants predisposing patients to myeloid neoplasms is critical for making treatment decisions, identifying at-risk family members, and selecting donors for hematopoietic stem cell transplantation [26]. However, the frequency of variants and spectrum of genes involved vary slightly among studies, and racial differences may contribute to these variations. In Western populations, the frequency of germline predisposition in patients with young-onset MDS is approximately 7–17% [13,14,15]. Keel et al. reported that 13.6% of 110 children and young adults (<40 years old) with MDS had a germline predisposition, with germline variants in *FANCA*, *MPL*, *RTEL4*, *SBDS*, *TERT*, *TINF2*, *GATA2*, *RUNX1*, and *TP53* [13]. Wlodarski et al. conducted a study targeting *GATA2* and found germline *GATA2* variants in 7% of 426 children and adolescents with *de novo* MDS, particularly in 15% of the patients with advanced MDS [14]. Moreover, Schwartz et al. found germline *SAMD9* or *SAMD9L* variants in 17% of 46 patients with *de novo* MDS [15]. In our study, a germline predisposition was identified in 16.1% of the patients with young-onset MDS, and the overall frequency was similar to that reported in previous studies [13,14,15]. However, the patients primarily harbored *GATA2* variants, and no patients with classical bone marrow failure syndrome (such as Fanconi anemia) were identified.

GATA2 deficiency, caused by heterozygous germline *GATA2* variants, is associated with MDS/AML, monocytopenia and mycobacterial infections (MonoMAC), dendritic cell, monocyte, B and natural killer (NK) lymphoid deficiency (DMLC), and/or lymphedema [9,28]. In a study reporting the natural history of GATA2 deficiency in French and Belgian patients, the median age at the first clinical symptom onset was 19 years [28]. MDS (70%) was the most common hematological malignancy, followed by cutaneous or genital recurrent HPV-related warts (40%) and severe bacterial infections (56%). In our study, *GATA2* variants were identified in 9.7% of patients, all of whom were men in their 20s, and the clinical manifestations related to GATA2 deficiency were heterogeneous among the patients. Studies have indicated a higher frequency of leukemia in patients with *GATA2* missense variants and an increased risk of lymphedema in those with null variants [28,31]. However, the genotype–phenotype correlation in GATA2 deficiency has not been clearly observed, and phenotypic heterogeneity has been reported even within families with the same variant [32].

PGM3 deficiency, a rare autosomal recessive congenital glycosylation disorder, is a subtype of the severe immunodeficiency caused by variants in *PGM3*. It is characterized by various clinical phenotypes, including leukopenia, severe neutropenia, T/B lymphopenia, skeletal dysplasia, and progression to bone marrow failure [33]. In this study, we found for the first time that PGM3 deficiency predisposes patients to MDS. In primary immunodeficiencies, the lack of effective immune surveillance against persistent and recurrent infections has been suggested to contribute to an increased risk of tumors and myeloid neoplasms [34,35]. Overall, in our study, primary immunodeficiencies, including GATA2 deficiency, were identified as the predominant factor, accounting for 12.9% of germline predispositions in young-onset MDS patients.

Few studies have reported somatic mutations focusing on patients with young-onset MDS [36,37]. In this study, we found *U2AF1* mutations in 29% of the patients, suggesting that these mutations are enriched in young-onset MDS. Conversely, mutations in *DNMT3A*, *ASXL1*, *SF3B1*, and *TP53*, which are typically prevalent in MDS and frequently occur during age-related hematopoiesis [7], were rarely observed in our study. *U2AF1* is a small subunit of U2 snRNP auxiliary factor (U2AF) that is involved in pre-mRNA processing. *U2AF1* mutations have been reported in 7–11% of patients with MDS, primarily involving codon S34 and Q157 mutations [37,38,39,40]. Although some studies have not shown a significant association between *U2AF1* mutations and patient age [6,39,40], a higher prevalence of *U2AF1* mutations in young patients has been reported in several studies [37,41,42]. Wu et al. reported that *U2AF1* mutations were observed at a frequency of 7.5% in 478 patients with MDS, with a notably higher prevalence of 13.2% in patients under the age of 40 years [37]. In a study by Kim et al. involving 152 Korean patients with MDS, the frequency of *U2AF1* mutations was 16%, which was higher than that in Western populations [41]. Furthermore, the S34F mutation was associated with younger onset. Similarly, Li et al. reported the presence of *U2AF1* mutations in 17% of 511 Chinese patients with MDS, with higher prevalence in young patients [42]. In particular, in patients aged 40 or younger, the frequency of *U2AF1* mutations slightly exceeded 20%. Given that *U2AF1* mutations have been suggested to be early initiating genetic events in MDS [39,42], our findings indicate that *U2AF1* mutations play a particularly important role in the pathogenesis of MDS, occurring at a younger age. Additionally, while previous studies have suggested a potential association between *U2AF1* mutations and trisomy 8 [41,42], we did not observe such an association in our study. Moreover, *U2AF1* mutations have been reported to be associated with poor prognosis in MDS [43,44]. In line with these findings, our study revealed a distinct difference in patient PFS and OS based on the presence of *U2AF1* mutations, indicating a significant adverse prognostic impact of *U2AF1* mutations, particularly in young-onset MDS.

Moreover, we identified a somatic *UBA1* mutation, which was the underlying cause of VEXAS syndrome (Vacuoles, E1 enzyme, X-linked, Autoinflammatory, Somatic), in one patient. VEXAS syndrome is a disease characterized by rheumatic and hematological features and was first described in 2020 [45]. The majority of patients exhibit characteristic clinical manifestations of inflammatory conditions, such as relapsing polychondritis, Sweet syndrome, and nodular vasculitis, with 25–50% of patients developing MDS [45,46]. It primarily occurs in males, and M41T, which was observed in our patient, is the most common mutation, followed by M41V/L. To date, limited data have been published on the treatment of VEXAS, particularly in cases related to MDS [47]. In this study, we observed an improvement in relapsing polychondritis and MDS-related findings following allogeneic hematopoietic stem cell transplantation.

Our study has several limitations. Because BM samples were used, there is a possibility that germline variants predisposing to myeloid neoplasms may have been masked by somatic genetic rescue, albeit rare. Furthermore, the sensitivity of the NGS testing performed in patients with young-onset MDS was lower than that of the testing used in the control group. Thus, we cannot rule out the possibility that low-burden somatic mutations might not have been detected in patients with young-onset MDS, resulting in a low somatic mutation rate.

## 5. Conclusions

To the best of our knowledge, this is the first comprehensive investigation of germline predisposition gene variants in Korean patients with MDS. The prevalence of germline predisposition to myeloid neoplasm in young-onset MDS was approximately 16%, largely associated with primary immunodeficiencies, and we have demonstrated the *PGM3* variants as the cause of germline predisposition for the first time. Furthermore, somatic *U2AF1* mutations associated with poor prognosis were observed in approximately one third of the patients, suggesting the possibility of a distinct subtype in young-onset MDS patients. However, additional validation studies in a multi-center cohort are necessary to confirm these findings.

## Figures and Tables

**Table 1:** Clinical and laboratory characteristics of 31 patients with young-onset MDS.

Characteristics	
Male, N (%)	19 (61%)
Age, N (%)	
0–20 years	9 (29%)
21–30 years	12 (39%)
31–40 years	10 (32%)
Complete blood count	
WBC, ×10^9^/L (IQR)	2.68 (1.85–3.74)
Hb, g/dL (IQR)	8.3 (7.2–9.9)
PLT, ×10^9^/L (IQR)	72 (39–174)
2016 WHO classification, N (%)	
cMDS, RCC	4 (13%)
MDS-SLD	4 (13%)
MDS-MLD	9 (29.1%)
MDS-RS-MLD	1 (3.3%)
MDS-EB1	7 (22.6%)
MDS-EB2	3 (9.7%)
MDS-U	3 (9.7%)
2022 WHO classification, N (%)	
cMDS-LB	4 (13%)
MDS-LB	6 (19.4%)
MDS-LB-RS	1 (3.3%)
MDS-h	10 (32.3%)
MDS-IB1	7 (22.6%)
MDS-IB2	2 (6.5%)
MDS-bi*TP53*	1 (3.3%)
Cytogenetic risk ^a^, N (%)	
Very good	0
Good	19 (61.3%)
Intermediate	9 (29%)
Poor	0
Very poor	3 (9.7%)
HSCT, N (%)	17 (54.8%)
Progression to higher grade, N (%)	7 (22.6%)
Dead, N (%)	7 (22.6%)

^a^ Cytogenetic risk was classified according to the revised international prognostic scoring system (IPSS-R): very good, -Y, del(11q); good, normal, del(5q), del(12p), del(20q), double including del(5q); intermediate, del(7q), +8, +19, i(17q), any other single or double independent clones; poor, −7, inv(3)/t(3q)/del(3q), double including −7/del(7q) complex (3 abnormalities); very poor, complex > 3 abnormalities. MDS, myelodysplastic neoplasm; IQR, interquartile range; WBC, white blood cell; Hb, hemoglobin; PLT, platelet; cMDS, RCC, childhood MDS, refractory cytopenia of childhood; MDS-SLD, MDS with single lineage dysplasia; MDS-MLD, MDS with multilineage dysplasia; MDS-RS-MLD, MDS with ring sideroblasts and multilineage dysplasia; MDS-EB, MDS with excess blasts; cMDS-LB, childhood MDS with low blasts; MDS-LB, MDS with low blasts; MDS-LB-RS, MDS with low blasts and ring sideroblasts; MDS-h, MDS, hypoplastic; MDS-IB, MDS with increased blasts; MDS-biTP53, MDS with biallelic *TP53* inactivation.

**Table 2:** Patients with MDS harboring causative germline variants predisposing to myeloid neoplasms and their clinical and laboratory information.

Case	Age, Years	Sex	BM Dx (2022 WHO)	Karyotype	Causative Germline Variant (VAF, %)	Inh	Other Clinical Findings	Progression	HSCT	Outcome (OS)
C13	3	F	cMDS-LB	46,XX[20]	*PGM3*:c.871+5G>A, p.? (100)	AR	6q14.1q14.3 microdeletion; microcephaly; leukopenia; eosinophilia; recurrent pneumonia	-	−	Expired (3 months)
A3	28	M	MDS-LB	46,XX[20]	*ETV6*:c.1075C>T, R359* (45.5)	AD	History of thrombocytopenia; recurrent lower leg cellulitis	AML (15 months after initial Dx)	+	Survive (163 months)
A15	22	M	MDS-h	47,XY,8[14]/46,XY[6]	*GATA2*:c.1082G>A, R361H (44.4)	AD	Tumorous skin growth; warts on nostrils	-	−	F/U loss (31 months)
A19	27	M	MDS-IB2	46,XY[20]	*GATA2*:c.1168_1170del, K390del (82.8)	AD	Prolonged perianal abscess and fistula and intra-abdominal abscess at cecectomy site for acute appendicitis; aggravated and uncontrolled pneumonia	AML (3 months after initial Dx)	+	Expired (3 months)
A20	28	M	MDS-IB1	50,Y,+1,der(1;7)(q10;p10),+3,+8,+9,+19[16]/46,XY[4]	*GATA2*:c.423_426del, Y141* (48)	AD	History of hypocellular marrow with leukopenia	-	+	Survive (22 months)

MDS, myelodysplastic neoplasm; Dx, diagnosis; cMDS-LB, childhood MDS with low blasts; MDS-LB, MDS with low blasts; MDS-h, MDS, hypoplastic; MDS-IB, MDS with increased blasts; AR, autosomal recessive; AD, autosomal dominant; VAF, variant allele frequency; Inh, inheritance; AML, acute myeloid leukemia; HSCT, hematopoietic stem cell transplantation; OS, overall survival; F/U, follow-up.

**Table 3:** Somatic mutations identified in patients with young-onset MDS.

Case	Sex	Age, Years	BM Dx (2022 WHO)	Karyotype	Somatic Mutation		VAF (%)
C9	M	10	cMDS-LB	46,XY[20]	*BCOR*	c.4009C>T; p.Q1337*	46.9
C15	M	19	MDS-IB1	47,XY,+13[12]/46,XY[8]	*ASXL1*	c.1934dup; p.G646Wfs	22.5
					*U2AF1*	c.101C>A; p.S34Y	39.6
					*ETV6*	c.1191_1195dup; p.R399Pfs ^b^	33.7
A3 ^a^	M	28	MDS-LB	46,XY[20]	*NRAS*	c.35G>A; p.G12D	41.9
					*U2AF1*	c.101C>T; p.S34F	51.9
A11	M	21	MDS-LB	46,XY,inv(9)(q32q34)[4]/46,XY[16]	*U2AF1*	c.101C>A; p.S34Y	42.4
A12	M	25	MDS-IB1	46,XY[18]	*U2AF1*	c.101C>T; p.S34F	50.4
A16	F	21	MDS-IB1	46,XX[20]	*IDH1*	c.394C>T; p.R132C	26.2
					*PHF6*	c.820C>T; p.R274*	45.5
					*MPL*	c.1774C>T; p.R592* ^b^	24.1
					*ETV6*	c.463G>A; p.D155N ^b^	38.9
A17	M	24	MDS-LB	46,XY,del(20)(q11.2q13.3)[19]/46,XY[1]	*U2AF1*	c.101C>T; p.S34F	42.0
A19 ^a^	M	27	MDS-IB2	46,XY[20]	*SF3B1*	c.2098A>G; p.K700E	46.2
					*U2AF1*	c.101C>A; p.S34Y	44.0
A20 ^a^	M	28	MDS-IB1	50,XY,+1,der(1;7)(q10;p10),+3,+8,+9,+19[16]/46,XY[4]	*STAG2*	c.2358+1G>A; p.?	30.4
O4	M	32	MDS-h	46,XY[20]	*U2AF1*	c.101C>T; p.S34F	36.6
					*PHF6*	c.820C>T; p.R274*	95.3
O14	M	38	MDS-LB	46,XY[20]	*UBA1*	c.122T>C; p.M41T	83.3
O17	F	30	MDS-LB-RS	46,XY[20]	*ASXL1*	c.1934dup; p.G646Wfs	47.1
					*U2AF1*	c.101C>A; p.S34Y	44.1
O18	M	34	MDS-bi*TP53*	46,XY,del(5)(q13q33),−7,der(10;12)(q10;q10),+12,add(14)(p11.2),−15,−20,+22,+22,+mar[11]/46,sl,del(3)(p21p25)[4]/46,XY[5]	*DNMT3A*	c.1628dup; p.R544Pfs	33.4
					*TP53*	c.455C>T; p.P152L	38.4
					*TP53*	c.817C>T; p.R273C	40.3
O19	M	38	MDS-IB1	46,XY[20]	*U2AF1*	c.101C>T; p.S34F	36.8

^a^ Patient carrying a causative germline variant predisposing to myeloid neoplasm. ^b^ Confirmed somatic mutation. MDS, myelodysplastic neoplasm; Dx, diagnosis; cMDS-LB, childhood MDS with low blasts; MDS-LB, MDS with low blasts; MDS-h, MDS, hypoplastic; MDS-IB, MDS with increased blasts; MDS-bi*TP53*, MDS with biallelic *TP53* inactivation; VAF, variant allele frequency.

**Table 4:** Comparison of major somatic mutations according to the onset age in patients with MDS.

Somatic Mutations		Young-Onset MDS (Age < 40 Years, N = 31)	Late-Onset MDS (Age ≥ 40 years, N = 50)	*p*-Value
*ASXL1*	N (%)	2 (7%)	9 (18%)	0.190
	Median VAF, % (range)	35 (23–47)	42 (15–49)	
*DNMT3A*	N (%)	1 (3%)	10 (20%)	0.044
	Median VAF, % (range)	33	26 (2–43)	
*SF3B1*	N (%)	1 (3%)	9 (18%)	0.080
	Median VAF, % (range)	46	25 (1–39)	
*STAG2*	N (%)	1 (3%)	3 (6%)	1.000
	Median VAF, % (range)	30	65 (37–93)	
*TP53*	N (%)	1 (3%)	13 (26%)	0.013
	Median VAF, % (range)	40	41 (10–94)	
*U2AF1*	N (%)	9 (29%)	3 (6%)	0.008
	Median VAF, % (range)	42 (37–52)	37 (3–44)	

MDS, myelodysplastic neoplasm; VAF, variant allele frequency.

## Data Availability

All data relevant to the study have been included in the article or uploaded as supplementary information. The additional data presented in this study are available upon request from the corresponding author.

## Supplementary Materials

The online version contains supplementary material available at https://www.mdpi.com/article/10.3390/jcm12247651/s1, Table S1. The gene list of the targeted NGS panel conducted in the control group (N = 38); Table S2: Target gene list for detecting germline variant predisposing to myeloid neoplasm in patients with young-onset MDS (N = 524); Table S3: Target gene list for somatic mutation detection in patients with young-onset MDS (N = 38); Table S4: Comparison of clinical and laboratory characteristics of 31 patients with young-onset MDS according to somatic *U2AF1* mutation status.
