# Supplementary material for: Genetic Characteristics of Patients with Young-Onset Myelodysplastic Neoplasms
Source: J Clin Med. 2023 Dec 13;12(24):7651. doi: 10.3390/jcm12247651 (PMC10743392; doi:10.3390/jcm12247651)
Supplement: Supplementary file 1 [file jcm-12-07651-s001.zip › jcm-2665179-supplementary.pdf]

Table S1. The gene list of the targeted NGS panel conducted in the control group (N=38)

Genes
ABL1, ASXL1, BCOR, CALR, CBL, CEBPA, CSF3R, DNMT3A, ETV6, EZH2, FLT3, GATA2, HRAS, IDH1, IDH2, IKZF1, JAK2, KIT, KRAS, MPL, NF1, NPM1, NRAS, PHF6, PRPF8, PTPN11, RB1, RUNX1, SETBP1, SF3B1, SH2B3, SRSF2, STAG2, TET2, TP53, U2AF1, WT1, ZRSR2

Table S2. Target gene list for detecting germline variant predisposing to myeloid neoplasm in patients with young-onset MDS (N=524)

Genes
ACD, ACP5, ACTB, ADA, ADAM17, ADAR, AICDA, AIRE, AK2, AKT1, ALAS2, ALK, ANKRD26, AP1S3, AP3B1, AP3D1, APC, APOL1, ARPC1B, ATM, ATP6AP1, ATR, B2M, BACH2, BCL10, BCL11B, BLM, BLNK, BLOC1S3, BLOC1S6, BMPR1A, BRAF, BRCA1, BRCA2, BRIP1, BTK, BUB1B, C15orf41, C1QA, C1QB, C1QC, C1R, C1S, C2, C3, C4A, C4B, C5, C6, C7, C8A, C8B, C8G, C9, CARD11, CARD14, CARD9, CARMIL2, CASP10, CASP8, CBL, CCBE1, CD19, CD247, CD27, CD3D, CD3E, CD3G, CD4, CD40, CD40LG, CD46, CD55, CD59, CD70, CD79A, CD79B, CD81, CD8A, CDAN1, CDC73, CDCA7, CDKN1C, CDKN2A, CEBPA, CEBPE, CFB, CFD, CFH, CFHR1, CFHR2, CFHR3, CFHR4, CFHR5, CFI, CFP, CFTR, CHD7, CHEK2, CIITA, CLCN7, CLPB, CNBP, COLEC11, COPA, CORO1A, CR2, CSF2RA, CSF2RB, CSF3R, CTC1, CTLA4, CTPS1, CTR9, CTSC, CXCR4, CYBA, CYBB, DCLRE1B, DCLRE1C, DDB2, DDX41, DICER1, DIS3L2, DKC1, DNAJC21, DNASE1L3, DNASE2, DNMT3B, DOCK2, DOCK8, DTNBP1, EFL1, ELANE, EPCAM, EPG5, ERCC1, ERCC2, ERCC3, ERCC4, ERCC5, ERCC6L2, ETV6, EXTL3, EZH2, F12, FAAP24, FADD, FANCA, FANCB, FANCC, FANCD2, FANCE, FANCF, FANCG, FANCI, FANCL, FANCM, FAS, FASLG, FAT4, FCGR1A, FCGR2A, FCGR2B, FCGR3A, FCGR3B, FCN3, FERMT3, FOXN1, FOXP3, FPR1, G6PC3, G6PD, GAD1, GATA1, GATA2, GFI1, GINS1, GLRX5, GP1BA, GPC3, GTF2H5, GUCY2C, H19, HAX1, HELLS, HMOX1, HPS1, HPS3, HPS4, HPS5, HPS6, HRAS, HTRA2, HYOU1, ICOS, ICOSLG, IFIH1, IFNAR2, IFNGR1, IFNGR2, IGF2, IGHG2, IGHM, IGLL1, IKBKB, IKBKG, IKZF1, IL10, IL10RA, IL10RB, IL12B, IL12RB1, IL17F, IL17RA, IL17RC, IL1RN, IL21, IL21R, IL2RA, IL2RG, IL31RA, IL36RN, IL6ST, IL7R, INO80, IRAK1, IRAK4, IRF2BP2, IRF3, IRF7, IRF8, ISG15, ITCH, ITGA2B, ITGAM, ITGB2, ITK, JAGN1, JAK1, JAK3, KCNN4, KDM6A, KLLN, KMT2A, KMT2D, KRAS, LAMTOR2, LAT, LCK, LIG1, LIG4, LPIN2, LRBA, LRRC8A, LYST, LYZ, LZTR1, MAGT1, MALT1, MAP2K1, MAP2K2, MAP3K14, MASP1, MASP2, MBL2, MCM4, MECOM, MEFV, MEN1, MKL1, MLH1, MOGS, MPI, MPL, MPO, MS4A1, MSH2, MSH6, MSN, MTAP, MTHFD1, MVK, MYD88, MYH9, MYO5A, MYO5B, MYSM1, NAF1, NBAS, NBN, NCF1, NCF2, NCF4, NCSTN, NF1, NF2, NFAT5, NFIX, NFKB1, NFKB2, NFKBIA, NHEJ1, NHP2, NLRC4, NLRP1, NLRP12, NLRP3, NOD2, NOP10, NOTCH3, NRAS, NSD1, NSMCE3, ORAI1, OSTM1, OTULIN, PALB2, PARN, PAX5, PAX6, PDGFRA, PDGFRB, PEPD, PGM3, PHOX2B, PIK3CA, PIK3CD, PIK3R1, PLCG2, PLEKHM1, PMS2, PNP, POLA1, POLE, POLE2, POLH, POMP, POT1, PPP1CB, PRF1, PRKAR1A, PRKCD, PRKDC, PSEN1, PSENEN, PSMA3, PSMB4, PSMB8, PSMB9, PSTPIP1, PTCH1, PTEN, PTPN11, PTPN22, PTPRC, RAB27A, RAC2, RAD51C, RAF1, RAG1, RAG2, RANBP2, RASGRP1, RB1, RBCK1, RBM8A, RC3H1, RECQL4, RELB, REST, RET, RFX5, RFXANK, RFXAP, RHOH, RIPK1, RIT1, RMRP, RNASEH2A, RNASEH2B, RNASEH2C, RNF168, RNF31, RNU4ATAC, RORC, RPL11, RPL15, RPL26, RPL27, RPL31, RPL35A, RPL5, RPS10, RPS19, RPS24, RPS26, RPS28, RPS29, RPS7, RPSA, RTEL1, RUNX1, SAMD9, SAMD9L, SAMHD1, SART3, SBDS, SDHB, SDHD, SEC23B, SEMA3E, SERPING1, SGPL1, SH2D1A, SH3BP2, SHOC2, SKIV2L, SLC19A2, SLC25A38, SLC29A3, SLC35C1, SLC37A4, SLC39A7, SLC46A1, SLX4, SMAD4, SMARCA4, SMARCAL1, SMARCB1, SMARCD2, SNX10, SOS1, SOS2, SP110, SPINK5, SPPL2A, SPRED1, SQSTM1, SRP54, SRP72, STAT1, STAT2, STAT3, STAT4, STAT5B, STIM1, STK11, STK4, STN1, STX11, STXBP2, SUFU, T, TAP1, TAP2, TAPBP, TAZ, TBK1, TBX1, TBXAS1, TCF3, TCIRG1, TCN2, TERC, TERT, TFRC, THBD, THPO, TICAM1, TINF2, TIRAP, TLR3, TMC6, TMC8, TMEM173, TNFAIP3, TNFRSF11A, TNFRSF13B, TNFRSF13C, TNFRSF1A, TNFRSF4, TNFSF11, TNFSF12, TP53, TPP2, TRAC, TRAF3, TRAF3IP2, TREX1, TRIM28, TRIM37, TRIP13, TRNT1, TSC1, TSC2, TTC37, TTC7A, TUBB1, TYK2, UBE2T, UNC119, UNC13D, UNC93B1, UNG, USB1, USP18, VHL, VPS13B, VPS45, WAS, WDR1, WIPF1, WRAP53, WRN, WT1, XIAP, XPA, XPC, XRCC2, ZAP70, ZBTB24, ZCCHC8, ZNF341

Table S3. Target gene list for somatic mutation detection in patients with young-onset MDS (N=38)

Genes
ABL1, ASXL1, BCOR, CALR, CBL, CEBPA, CSF3R, DNMT3A, ETV6, EZH2, FLT3, GATA2, HRAS, IDH1, IDH2, JAK2, KIT, KRAS, MPL, NF1, NPM1, NRAS, PHF6, PRPF8, PTPN11, RB1, RUNX1, SETBP1, SF3B1, SH2B3, SRSF2, STAG2, TET2, TP53, U2AF1, UBA1, WT1, ZRSR2

Table S4. Comparison of clinical and laboratory characteristics of 31 patients with young-onset MDS according to somatic *U2AF1* mutation status

	U2AF1 mutation (-) (N=22, 71%)	*U2AF1* mutation (+) (N=9, 29%)	*p*-value
Male, N (%)	11 (50%)	8 (89%)	*0.101*
Age, N (%)			
0–20 years	8 (36%)	1 (11%)	*0.329*
21–30 years	7 (32%)	5 (56%)	
31–40 years	7 (32%)	3 (33%)	
Complete blood count			
WBC, $\times 10^9$/L (IQR)	2.73 (1.99–3.56)	2.32 (1.83–5.13)	*1.000*
Hb, g/dL (IQR)	8.6 (7.4–10)	8.1 (6.9–9.9)	*0.446*
PLT, $\times 10^9$/L (IQR)	57.5 (35–141)	142 (52–174)	*0.139*
WHO classification, N (%)			
MDS-SLD/MLD/Others	16 (73%)	5 (56%)	*0.588*
MDS-EB1	4 (18%)	3 (33%)	
MDS-EB2	2 (9%)	1 (11%)	
Cytogenetic risk classification[a], N (%)			
Good	12 (55%)	7 (78%)	*0.596*
Intermediate	7 (32%)	2 (22%)	
Poor	0	0	
Very poor	3 (14%)	0	
HSCT, N (%)	10 (45%)	7 (78%)	*0.106*
Progression to higher grade, N (%)	2 (9%)	5 (56%)	*0.012*
Death, N (%)	2 (9%)	5 (56%)	*0.012*

[a]Cytogenetic risk was classified according to the revised international prognostic scoring system (IPSS-R): very good, -Y, del(11q); good, normal, del(5q), del(12p), del(20q), double including del(5q); intermatiate, del(7q), +8, +19, i(17q), any other single or double indepencent clones; poor, -7, inv(3)/t(3q)/del(3q), double including -7/del(7q) complex (3 abnormalities); very poor, complex > 3 abnormalitieis.

MDS, myelodysplastic neoplasm; IQR, interquartile range; WBC, white blood cell; Hb, hemoglobin; PLT, platelet; MDS-SLD, MDS with single lineage dysplasia; MDS-MLD, MDS with multilineage dysplasia; MDS-EB, MDS with excess blasts; HSCT, allogeneic hematopoietic stem cell transplantation
